# The Impact of Three‐Dimensional Printed Anatomical Models on First‐Year Student Engagement in a Block Mode Delivery

**DOI:** 10.1002/ase.1958

**Published:** 2020-04-16

**Authors:** Nicholas Tripodi, Kate Kelly, Maja Husaric, Rebecca Wospil, Michael Fleischmann, Susan Johnston, Katherine Harkin

**Affiliations:** ^1^ First Year College Victoria University Melbourne Victoria Australia; ^2^ Osteopathy Division College of Health and Biomedicine Victoria University Melbourne Victoria Australia

**Keywords:** gross anatomy education, undergraduate education, student engagement, practical assessments, 3D‐printing, block mode, intensive teaching

## Abstract

Student engagement is known to have several positive effects on learning outcomes and can impact a student's university experience. High levels of engagement in content‐heavy subjects can be difficult to attain. Due to a major institutional restructure, the anatomy prosection laboratory time per subject was dramatically reduced. In response, the authors set out to redesign their anatomy units with a focus on engaging the learning activities that would increase time‐on‐task both within and outside of the classroom. One of these curriculum changes was the implementation of a suite of anatomy learning activities centered on sets of three‐dimensional printed upper limb skeleton models. A two‐part mixed‐method sequential exploratory design was used to evaluate these activities. Part one was a questionnaire that evaluated the students' engagement with and perceptions of the models. Part two involved focus groups interviews, which were an extension of the survey questions in part one. The results of the study indicated that the majority of students found the models to be an engaging resource that helped improve their study habits. As a result, students strongly felt that the use of the models inspired greater academic confidence and overall better performance in their assessments. Overall, the models were an effective way of increasing the engagement and deep learning, and reinforced previous findings from the medical education research. Future research should investigate the effects of these models on student's grades within osteopathy and other allied health courses.

## INTRODUCTION

Student engagement is critical in student learning, success, and retention at a tertiary level (Zepke and Leach, 2010a; Kahu, 2013; Northey et al., 2015) and subsequently, has been a major focus of higher education pedagogical research for a number of years (Krause and Coates, 2008; Wilson et al., 2018). Student engagement is a complex and multifaceted aspect of the classroom experience with no definitive and holistic definition that unifies all its varying components (Zepke and Leach, 2010b; Kahu, 2013). Despite its fluid and context‐dependent definition (Zepke and Leach, 2010b), student engagement can be broadly conceptualized as the time and physical energy that students expend on activities in their academic experience (Robinson and Hullinger, 2008). Kahu (2013) describes the student engagement across four distinct domains: behavioral; psychological; socio‐cultural; and holistic. Specifically, the behavioral domain refers to how student behavior and teaching practices relate to engagement. The psychological domain describes the engagement as incorporating characteristics such as motivation, self‐determinism, and expectations. The socio‐cultural domain acknowledges impacts of the broader social context, including influences from society, culture, and the historical context of the classroom. Finally, the holistic domain aims to draw the three other aspects together and contextualize how engagement in these areas extend beyond the classroom.

A student's approach to learning is defined as “a way in which students go about their academic tasks, thereby effecting their learning outcomes” and can directly influence their performance and success within a subject (Biggs, 1994; Azer et al., 2013). A deep learning approach implies a more profound conceptualization of knowledge, which includes the ability to apply, elaborate, and analyze the content (Biggs, 1994; Aharony, 2006; Azer et al., 2013). Deep learning strategies in anatomy education are important because they correlate positively to the quality of learning (Pandey and Zimitat, 2007). Deep learning can be effectively implemented by encouraging students to have a primary role in the construction of knowledge they seek (Azer et al., 2013). More specifically, in anatomy education, deep learning can be achieved through learning strategies such as visualization, self‐directed learning, discussion with others, and clinical application (Pandey and Zimitat, 2007; Findlater et al., 2012; McLean, 2016). This is juxtaposed with surface learning, where the facts or content are memorized on a superficial level, without any real‐world purpose or application (Biggs, 1994; Aharony, 2006; Pandey and Zimitat, 2007). Surface learning is a common learning approach in anatomy, whereby the educator is often seen as the presenter of knowledge and students as the consumers (Pandey and Zimitat, 2007; Findlater et al., 2012). However, it should be noted deep and surface learning strategies can be used synergistically in this setting, as rote learning and memorization is often a component of conceptualizing the anatomical knowledge in a deeper way (Pandey and Zimitat, 2007). When students are engaged in the content, they show a propensity for deeper learning and tend to intrinsically value their course more highly. In addition, students who primarily use a deep learning approach report higher perceived academic confidence and greater overall satisfaction with their tertiary education (Carini et al., 2006; Laird et al., 2008). Conversely, students who are less engaged tend toward a surface learning approach (Floyd et al., 2009). When students are disengaged in the classroom and from the content, research clearly indicates that this often results in poorer outcomes and a lower perceived course value (Carini et al., 2006; Laird et al., 2008; Floyd et al., 2009; Kraus et al., 2011).

In recent years, three‐dimensional printing (3DP) has emerged as an innovative teaching tool to enhance the anatomical education (Estai and Bunt, 2016). The procedural advantages of 3DP include cost efficiency and both ethical and legal advantages in comparison to the traditional teaching methods such as cadaver prosection/dissection, preserved bones, and plastic models (AbouHashem et al., 2015; Fredieu et al., 2015). The use of models to assist in educating students in anatomy is less likely to exclude students who may be unable to engage in cadaver training due to religious or cultural beliefs, or those who have difficulties accessing the online resources due to the complexity of many information and communication technology tools (Lockwood and Roberts, 2007). Many tertiary institutions are moving away from or completely abandoning cadaver‐based teaching, mostly due to the increasing cost to obtain, dissect, and maintain the cadavers (McMenamin et al., 2014; Losco et al., 2017; Wilson et al., 2019). In addition, a large proportion of undergraduate students do not require advanced knowledge of the entire body to achieve the learning outcomes and accreditation requirements of their course, thus 3DP models can be an appropriate alternative and hence, are becoming increasingly common within the classroom (McLachlan and Regan De Bere, 2004).

Research has shown that students using the 3DP anatomical models demonstrate equal or better test scores compared to students who engaged with the more traditional anatomy teaching tools such as computer programs and cadavers (Preece et al., 2013; Li et al., 2015; Lim et al., 2016). Furthermore, 3DP anatomy models may enhance or complement other forms of learning, which as previously established, enhances both deep learning and engagement among students (Estai and Bunt, 2016). Literature also suggests that that the quality and detail of the 3DP model can aid in achieving higher degrees of successful learning outcomes (Kong et al., 2016). More recently, it has been shown that personal 3DP anatomical models can be used effectively to augment both the curriculum and more traditional laboratory‐based learning approaches (Mogali et al., 2018; Smith et al., 2018; Backhouse et al., 2019). Despite the widespread adoption of 3DP anatomical models into medical and surgical education and training (Baskaran et al., 2016; Fasel et al., 2016; Lim, et al., 2016), there is little research that explores the effectiveness of their use in other health education disciplines (Azer and Azer, 2016).

In 2018, Victoria University (Melbourne, Australia) underwent a transformational change that saw the removal of traditional lectures from all first‐year units. This change was continued across all undergraduate study years in 2019. At the time of evaluation, all first‐year subjects were delivered in a block‐mode format, that consisted of three, three‐hour workshops each week (with slight variations in contact hours between subjects), staffed by the same teacher. Students complete each of their four‐week units individually, with four units completed per semester. This change compelled the authors to redesign their anatomy learning activities to have a higher impact on student engagement in courses that have poorer student retention and performance, as this has been shown to be pivotal in increasing both the student and institutional success (Zepke and Leach, 2010a). Furthermore, as a result of this change, time available with the cadaver laboratory became more limited. The teaching faculty also carefully considered the different student personalities, backgrounds, learning styles, and pedagogical approaches when designing these 21^st^ Century learning activities, to best suit this cohort of modern students (DiLullo et al., 2011).

### Research Aims

To this end, the authors set out to examine if a series of interactive learning activities based around a set of in‐house printed 3D bones could broadly appeal to and increase the engagement of first‐year osteopathic students in block‐mode delivery. Furthermore, this research aimed to qualitatively explore if the use of 3DP models in high‐fidelity environments impacted perceived student confidence and anxiety levels related to their assessments. Given the positive effect of 3DP anatomical models previously reported in medical education, this article also aims to confirm these results in an alternate student cohort.

## MATERIALS AND METHODS

### Study Design and Evaluations

A two‐part mixed‐method sequential exploratory design was used. Part one was a questionnaire that evaluated the students' usage, engagement, and perceptions of the 3DP anatomical models and associated learning activities. As there was no suitable questionnaire developed in the literature, all items on the questionnaire were adapted from analogous research in the allied health literature and osteopathic education (Weeks and Horan, 2013; Tripodi, 2018). The survey was a five‐point Likert‐type scale survey that ranged from 1 = strongly disagree to 5 = strongly agree and consisted of seven items related to the students’ use, perceptions, and engagement with the 3DP models (Table 1). The survey also contained four open‐ended questions exploring similar themes to the Likert‐type items. These additional questions were designed to obtain additional qualitative data and to help ensure data saturation was reached (Appendix). Part two consisted of focus group interviews. The focus group interviews were conducted by one of the study authors and guided by a set of questions that were an extension of the original survey (Appendix). The focus group interviews were 15–20 minutes in duration. Both the surveys and focus group interviews were conducted 1 week after the completion of the unit, in June 2018.

**Table 1:** The Students’ Self‐Reported Use, Perceptions, and Engagement with the Three‐Dimensional Printed Models

Statement	Number of Responses; N	Responses; Mean (±SD)	χ^2^ (df = 2)	*P*‐value
Helped me review material from class	111	4.57 (±0.68)	193.02	<0.001
Helped me prepare for the anatomy *viva* assessment	111	4.46 (±0.85)	150.97	<0.001
Allowed me to learn independently	111	4.36 (±0.89)	141.46	<0.001
Motivated me to study	111	3.95 (±0.99)	65.08	<0.001
Reduced my need to take notes	111	3.39 (±1.14)	6.70	<0.05
Improved my anatomy *viva* assessment performance	111	4.26 (±0.87)	113.08	<0.001
Overall, improved my learning experience and performance in this unit	111	4.40 (±0.79)	156.32	<0.001

Each response was rated on a five‐point Likert scale ranging from 1 = strongly disagree to 5 = strongly agree. Responses were categorized as positive (somewhat agree, strongly agree), neutral (neither agree nor disagree), and negative responses (somewhat disagree, strongly disagree). Chi‐Square Goodness‐of‐Fit Test (χ^2^) demonstrated a statistically significant skew toward positive responses in all questions, except for reducing the need to take notes, which was mainly neutral. *Viva* refers to the final laboratory‐based oral anatomy examination.

### Participants

First‐year osteopathy students who were enrolled in the unit Scientific Basis for Osteopathy 1 (SBO1) in semester 1, 2018 were asked to participate in this project via email. A total of 145 students were contacted and from that 111 (n = 111) participants completed the survey representing a 77% response rate. Furthermore, eight (n = 8) students took part in the focus groups (6%). There were two separate focus groups, one group consisted of five (n = 5) students, the other three (n = 3) students. The mean age of the participants was 20 years old. About 59 (53.2%) participants had studied anatomy before, 52 had not (46.8%). About 90 participants (81.1%) had not completed any postsecondary study, while 21 (18.9%) had. English was the first language for 108 participants (97.3%), while for three participants (2.7%) it was not. All of the participants were domestic students.

### Participant Recruitment

The students were informed of the present study through an email at the beginning of the unit, in May 2018. The email contained a link to the “Information to Participants” form and after this, a link to the survey. The survey was conducted using Qualtrics (Provo, UT). The consent information was presented to the participants prior to the survey. The survey was then made accessible if the participants consented. Students who were interested in participating in the focus groups were asked to contact the principle researcher via email. Consent for the focus groups was gained via a separate consent form.

### Institutional and Unit Setting

**Figure 1:**
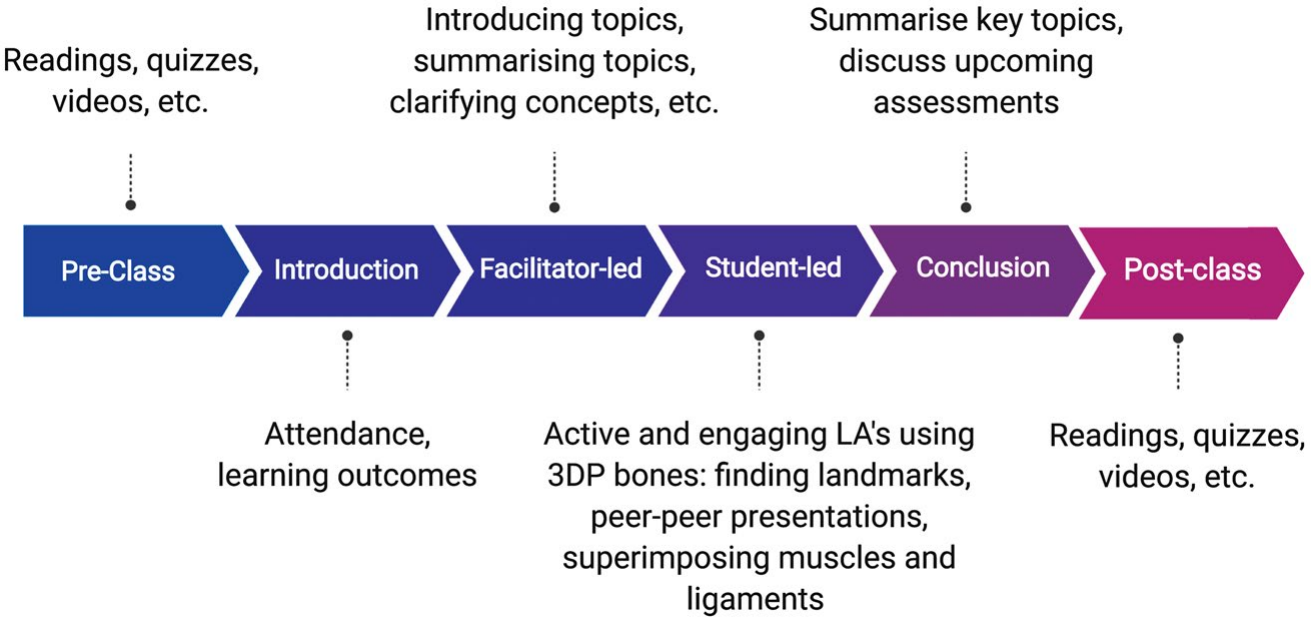
Schematic diagram of the Scientific Basis for Osteopathy 1 (SBO1) class structure in the Victoria University's block mode. Classes are divided into six key phases: pre‐class, introduction, facilitator‐led, student‐led, conclusion, and post‐class. Emphasis is placed on pre‐class activities that facilitate a deeper style of learning; 3DP, three‐dimensional printing; LA, learning assessments.

At the time the study took place, SBO1 underwent a significant redesign to conform to Victoria University's “block model” delivery. SBO1 is a unit consisting of anatomy, biomechanics, and physiology applied to the upper limb (Fig. 1). SBO1 ran in semester one, May, 2018. Students had 18 compulsory contact hours per week for the unit. Three of these weekly hours take place in the anatomy laboratory (9 hours total for the unit), where the learning activities are based around prosected cadavers, bony specimens, and full‐size plastic anatomical models. All the new unit content and laboratory sessions are delivered in the first 3 weeks, with the final assessments taking place in the fourth week. Students were assessed via two theory‐based quizzes (week 2 and week 4), three case‐based leaning assessments (weeks 1–3), and a final laboratory‐based oral anatomy examination in week 4 (*viva*). The *viva* comprised of the student presenting three anatomy specimens to the examiner within a 15‐minute period, and was a hurdle requirement worth 25% of their final grade. The three specimen categories were a bone, a muscle, and a nerve pathway of the upper limb.

### Learning Activities

**Figure 2:**
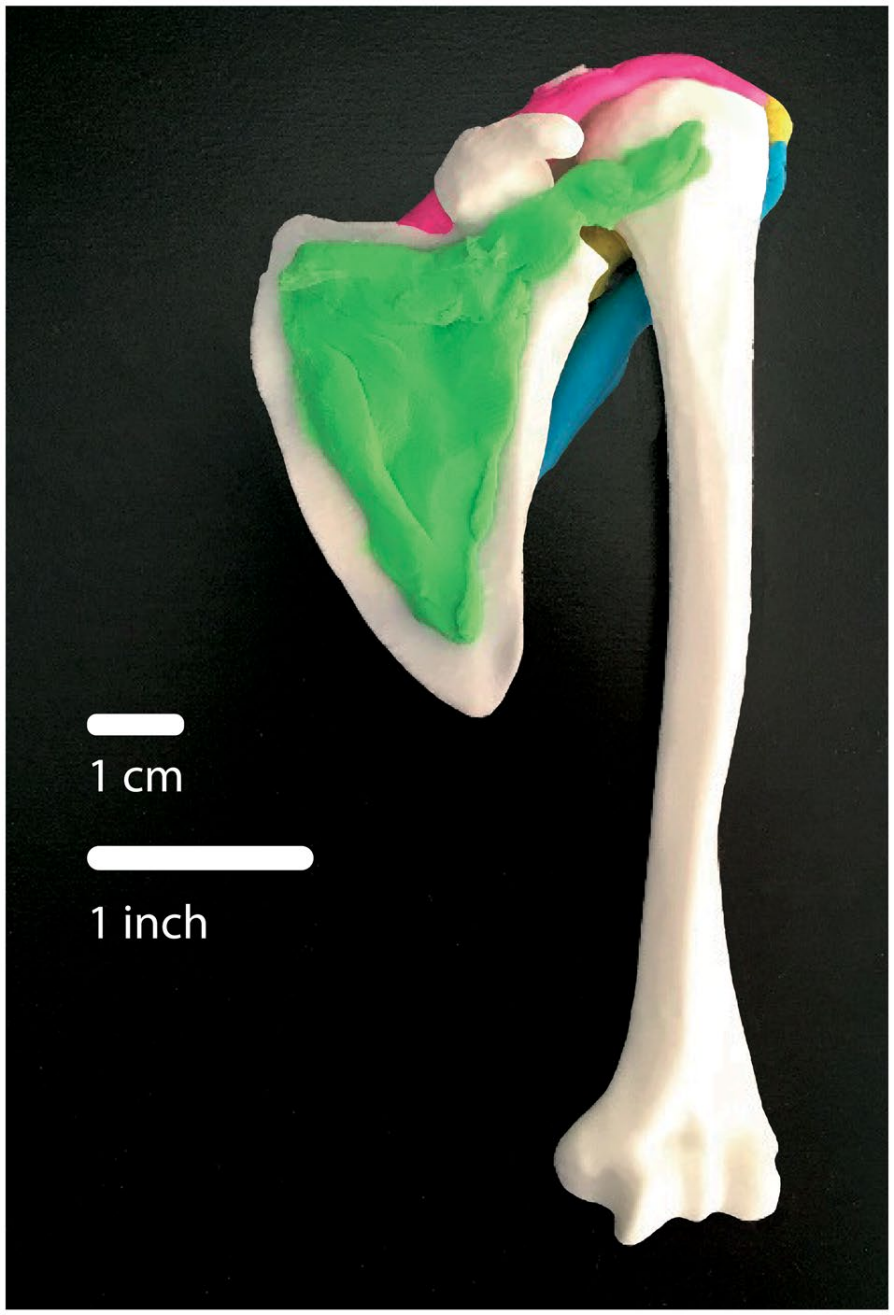
An example of the three‐dimensional models that were made available to the students. Students received a clavicle, scapula, humerus, radius, ulna, carpal and metacarpal bones, and phalanges. The models were printed on a Flashforge Inventor II 3D printer using polylactic acid (PLA) bioplastic polymer with nozzle diameter 0.4 mm and a layer resolution of 0.18 mm. Muscles overlaying bones and joints are made of reusable putty‐like adhesive.

Given the low cost incurred from printing the models in‐house, students were each given a set of 3DP bones of the upper limb to keep (scapula, clavicle, humerus, ulna, radius, carpal and metacarpal bones, and phalanges). The students completed approximately 4 hours of in‐class activities with the bones directly each study week. These activities consisted of anatomical orientation, identifying bony landmarks, using Blu‐Tack Color reusable putty‐like adhesive (Bostik, Stafford, England) to form and overlay muscles on bones and joints (Fig. 2), followed by mock peer‐to‐peer presentations. The 3DP bones were used to augment the loss of laboratory time, with a heavy focus on preparing students for their final oral anatomy assessment. Students were also encouraged to repeat the in‐class activities with the 3DP bones at home for a similar time that they spent on the activities in class.

### Three‐Dimensional (3D) Printing Process

The models were printed on a Flashforge Inventor II 3D Printer (Flashforge, Kowloon Bay, Hong Kong) using polylactic acid (PLA) bioplastic polymer. Each printer cost 635.15 ($USD) and the cost of filament was 1.25 ($USD) per model set. The PLA filament was 1.75 mm in diameter. The nozzle diameter was 0.4 mm and the layer resolution was 0.18 mm. The designs were prepared for printing using FlashPrint, version 3.22 (Flashforge, Kowloon Bay, Hong Kong) and Autodesk Meshmixer, version 3.5 (Autodesk, San Rafael, CA) software. The models were printed at a scale of 1:4, as to increase productivity time and reduce costs. Each set took approximately eight hours to print. All models were visually assessed by the teaching team to ensure anatomical accuracy.

### Data Analysis

Quantitative data were analyzed using SPSS statistical package, version 25 (IBM Corp., Armonk, NY). Descriptive statistics were generated for each questionnaire item. Questionnaire responses were categorized as positive (somewhat agree, strongly agree), neutral (neither agree nor disagree), and negative responses (somewhat disagree, strongly disagree). A Chi‐Square Goodness‐of‐Fit test was used to assess the differences in expected and observed response frequencies. Cronbach's alpha was used to evaluate the internal consistency of the Likert‐type scale questions (α = 0.953) showing that there was high internal consistency and reliability of the survey.

The focus group interviews were recorded on an electronic recording device then manually transcribed. The focus group transcriptions and the long‐answer survey questions were analyzed using the qualitative data analysis software NVIVO, version 11 (QSR International, Melbourne, Victoria, Australia). Using a thematic analysis framework, the qualitative data were broadly divided into behavioral, psychological, socio‐cultural, and holistic themes (Kahu, 2013) using a deductive approach. From here, multiple sub‐themes were generated and subsequently reviewed. This process was performed individually by three of the authors. The final thematic count was internally validated via group consensus based on previously described methods (Vaismoradi et al., 2013). Briefly, this method consisted of data familiarization first, followed by searching for and reviewing themes, then defining themes, and finally by report production.

### Ethical Approval

Ethical approval was obtained from the Victoria University Human Research Ethics Committee under the Victoria University First‐Year‐Model block ethics application (Approval Number: HRE 17‐192).

## RESULTS

### Quantitative Survey: Student Use, Perceptions, and Engagement

The participants reported a high level of use, engagement, and overall benefit when asked about their thoughts on the 3DP models (Table 1). Using a Chi‐Square Goodness‐of‐Fit Test all survey answers were found to be strongly positively skewed (*P < *0.001; Table 1), barring the reduced need to take notes, which was moderately skewed toward a positive response (χ^2^_(2)_ = 6.70, *P < *0.05; Table 1). The majority of students perceived the 3DP bones to be useful for both reviewing materials from class and to prepare for the *viva* (95% and 88.3% positive, respectively). Students also found the 3DP bones to be helpful for independent learning and for study motivation (86.5% and 67.5% positive, respectively). Finally, most students reported that the 3DP bones improved their *viva* performance and had a positive effect on the student learning experience and performance in the unit (80.1% and 89.2% positive, respectively).

### Focus Group and Long‐Answer Survey Findings

#### Theme 1: Behavioral factors

Behavioral factors were the most common theme identified in the qualitative analysis (52.5% of the total coded response rate). Students reported that the use of the 3DP bones improved their performance in the unit, and that using the 3DP bones allowed them to achieve higher results than anticipated. Using the 3DP bones at home, the palpable similarities to real life structures, and using the bones to simulate the *viva* were some of the key factors reported by students in improving their performance. Students reported that performing mock examinations prior to the *viva* with the 3DP bones was instrumental in both preparing for the *viva* and improving their performance during the *viva*. In terms of engagement, many students found the 3DP bones increased their levels of engagement with the class activities, and the content from the unit. The tactile and kinesthetic nature of the 3DP bones were key factors in increasing engagement. The ability to study with the bones outside of the university and class time provided the students with more autonomy for their own learning (Table 2).

**Table 2:** Focus Group and Long‐Answer Survey Findings; Theme 1: Behavioral Factors

Sub‐Themes	Coded Responses N (%)	Sample of Student Responses
Unit Performance	49 (13.4)	“I know that without the bones I may not have gotten as high a score as what I did.” “It gave me a greater perspective and therefore benefited my results and performance.”
*Viva* Performance	52 (14.2)	“The 3D bones helped me to have a visual representation of the bony landmarks on each bone which made it easier for me to find in the Viva assessment.” “I used the 3D bones to test myself as well as practicing my language use and verbal skills in relation to the oral exam.”
Student Engagement	71 (19.4)	“The bones enabled me to study outside of class with a visual representation that was in my hands and not on a computer.” “Looking at and touching models of bones feels like I learn about them in a more effective way than just reading some text or looking at pictures.”
Independent Learning	20 (5.5)	“Independent study at home allowed myself to distinguish different bone landmarks.” “It was easy because they fitted in my bag to do study anywhere.”

#### Theme 2: Psychological factors

The learning activities appear to have an effect on the identified psychological factors and accounted for 32.5 % of the total coded response rate. Students reported that the 3DP bones increased their confidence levels in preparation for their assessments. Students reported the main reasons for this increased confidence were due to the ability to study autonomously and the ability to feel and identify key structural landmarks on each bone prior to the *viva*. Some students (9.6%) also commented that using the 3DP bones led to a reduction in their anxiety levels for the *viva*. Although this was not reported as often as increased confidence, students said that were able to prepare better for the assessments, which reduced their anxiety levels. Students commented that owning their own bones allowed them to interact with the bones in ways in which they could not otherwise, such as drawing key landmarks on them and being able to study with them at any time (Table 3).

**Table 3:** Focus Group and Long‐Answer Survey Findings; Theme 2: Psychological Factors

Sub‐Themes	Coded Responses N (%)	Sample of Student Responses
Perceived Confidence	77 (21.0)	“Gave me more confidence to revise independently.” “It increased my confidence dramatically before my assessment.”
Perceived Anxiety	35 (9.6)	“It helped reduce my anxiety and stress as I was more prepared.” “Lowered my levels of anxiety as I wasn't as nervous going into the assessment as I had covered it at home on the 3D bones.”
Ownership	7 (1.9)	“Didn't need to go to uni in order to borrow a skeleton to study with.” “I liked having my own set of bones.”

#### Theme 3: Socio‐cultural factors

The themes related to socio‐cultural factors within the classroom accounted for 4.4% of the total coded response rate. Students commented how the 3DP bones were versatile to use and effective as they were tactile, could be drawn on, and used to help identify important anatomical landmarks. Students also observed that the 3DP bones were time effective compared to study with other material such as slides or images (Table 4).

**Table 4:** Focus Group and Long‐Answer Survey Findings; Theme 3: Socio‐Cultural Factors

Sub‐Themes	Coded Responses N (%)	Sample of Student Responses
Effective Learning Tool	16 (4.4)	“They are a good learning tool that should be utilized more throughout the course.” “I hope we get to keep using 3D bones as a learning tool.”

#### Theme 4: Holistic factors

The themes related to holistic factors within the classroom accounted for 10.4% of the total coded response rate. Students identified the benefits of using the 3DP bones as a learning tool for other subjects such as biomechanics and clinical skills, and for study in future years of anatomy to enhance the learning experience. Furthermore, students identified that there could be future improvements to the 3DP bones. They identified the quality of the 3DP bones, the size of the carpal bones, and orientation of the bone as areas of further improvement (Table 5).

**Table 5:** Focus Group and Long‐Answer Survey Findings; Theme 4: Holistic Factors

Sub‐Themes	Coded Responses N (%)	Sample of Student Responses
Assistance Beyond the Unit	6 (1.6)	‘I thought they were really helpful when we were learning the biomechanics.’ ‘They should be used for further anatomy subjects seeing that the technology is there to assist us in better learning and understanding.’
Future Improvements	32 (8.8)	“I would find it helpful if the bones where printed as right side bones, as most of our labelled resources show the left side.” “Better modelling to differentiate the landmarks.”

## DISCUSSION

Despite many studies finding positive effects for the use of 3DP anatomical models from a learning and procedural standpoint in medical education, little contemporary research has investigated these effects in the allied health or other health science areas (Azer and Azer, 2016). The aim of this project was to investigate if the benefits of 3DP anatomical models would be transferable to a cohort of first‐year osteopathic students undertaking block‐mode delivery, and therefore, confirm previous findings from medical education research.

The overarching results of this research indicated that the 3DP anatomical model learning activities were successful in increasing engagement of the students within a block‐model context. Previous studies almost unanimously show that 3DP anatomical models are effective in augmenting learning, specifically, in improving on grades and learning outcomes (Preece, et al., 2013; Li et al., 2015; Estai and Bunt, 2016; Lim et al., 2016; Chen et al., 2017; Garas et al., 2018; Wilson et al., 2019). Students reported that the 3DP anatomical model learning activities were engaging, fun and specific to their assessments. Higher levels of student engagement in first‐year students have been shown to enhance student learning outcomes and success by increasing GPA and increasing the likelihood of transitioning to second‐year (Kuh et al., 2008). It can also enhance personal and social development (Zhao et al., 2005) and may act as a conduit to develop students’ social and cultural capital, thus enhancing skills that apply beyond the workplace (Zepke and Leach, 2010b). Although this study did not measure any direct effects of the learning activity on grades, it is proposed that the effects on engagement played a strong role in students’ reporting an improved experience and performance in the unit.

The participant's responses were mostly positive to the survey items enquiring about the models being helpful in reviewing class material, helping to prepare for the *viva* assessment, promoting independent learning and improving study motivation. This indicates that in terms of psychological factors (Kahu, 2013) the learning activities promoted student engagement in the unit content more broadly and encouraged an active learning environment. These findings were also mirrored in the behavioral factors section of the thematic analysis. Heutagogy is a foundational learning theory where the guiding principle involves the appointment of the student as the principle director of their own learning (Amadieu et al., 2009). Much like Kahu's (2013) psychological and behavioral domains, heutagogy encourages self‐determinism within students and implores them to take control of their own knowledge acquisition (Amadieu et al., 2009). It involves a level of knowledge construction by the student that is facilitated by the educator. The behavioral, psychological, and socio‐cultural qualitative responses illustrated that students were able to use the bones as a knowledge construction tool, in particular for assisting in their understanding of the spatial relationship between the anatomical structures, conceptualizing upper limb biomechanics, and subsequently applying it to their case‐based learning assessments. This approach to learning allowed students to develop confidence constructing their own understanding of the structure and function, in turn empowering them and resulting in higher levels of perceived academic performance and confidence.

In the context of both this study, and the current higher education landscape, it is important to consider the intrinsic link between the engagement and perceived educational value. Students' perception of their course educational value is hard to measure (Alves, 2011), but is proposed to be a function of personal relevance and task meaningfulness, and is critical for students' overall learning experience (Floyd et al., 2009). Course value is said to be improved though experiences such as: a sense of learning community, students knowingly reaching their educational goals and more active and learner‐centered activities (Zhao and Kuh, 2004; Floyd et al., 2009; Petruzzellis and Romanazzi, 2010). High course value can promote student engagement, facilitate deep learning and improve overall learning outcomes (Floyd et al., 2009; Duque, 2014). The findings of this study demonstrate not only a high engagement with the learning activity and content, but also appears to have added value to the overall learning experience, through active and deep learning. Furthermore, the focus group findings mirror that of Backhouse et al. (2019), who found that a cohort of optometry using their own 3DP skull models to study anatomy strongly resonated with the ownership and personalized aspects of the models. Hence, the findings suggest that the concept of ownership is important in building student engagement and that these concepts may have a downstream effect on both student learning strategies and perceived course educational value.

Larger class sizes have been shown to correlate to a decrease in both teacher and student perceived student learning in higher education (Chapman and Ludlow, 2010) and also with a decrease in student performance and satisfaction (Cuseo, 2007; Kokkelenberg et al., 2008). It is also argued that the traditional lecture delivery models at universities are outdated and disengaging for the modern student (Kokkelenberg et al., 2008). Given the results of the present study, it is reasonable to assume that the replacement of lectures with smaller workshops and engaging learning activities in the first year of study fosters an improved perception of student learning and assists students in improving their academic performance. However, it cannot be definitively said what ratio that the positive experiences reported by the students were due solely to the learning activities, as the change to block‐mode delivery was not quantitatively evaluated here. Despite this, and considering results in other student cohorts (Mogali et al., 2018; Backhouse et al., 2019), it can be confidently said that 3DP anatomical models can augment anatomy education in both a “traditional” semester model and in block‐model delivery.

### Limitations of the Study and Future Directions

The predominate limitation of this study was that there was no control group to objectively demonstrate if the reported positive attitudes toward the 3DP learning activities could be causally linked to overall grades and performance. The study also did not control for the effect of institutional changes on the quantitative and qualitative responses. Further limitations include that this study only tested musculoskeletal anatomy, and as it took place at only one institution, generalizations to other curriculums should be applied with caution. In addition, some of the authors were part of the teaching team and it may have been pertinent to use a social desirability scale to account for any response bias (Van de Mortel, 2008). However, given the surveys and focus group interviews occurred after the unit had been completed, the authors believe that social desirability did not influence participant responses. Future research should aim to study the effect of 3DP models on student engagement and correlate the effects of the 3DP learning activities and models to assessment performance and student grades across other non‐medical disciplines.

## CONCLUSION

The results from this study indicate that the 3DP models and their associated learning activities are effective in increasing student engagement in first‐year osteopathy anatomy classes. It is proposed that the increased engagement was the result of student empowerment as they navigated the content in a self‐deterministic manner, constructing their own deeper knowledge. As students were taking responsibility for their own learning, as opposed to being content consumers, they undertook greater ownership, and this led to increased academic confidence and perceived performance. The results of this project show promise. First, they confirm existing findings that purport that the use of models in anatomy education as an engaging tool that aids in knowledge construction (McMenamin et al., 2014; Losco et al., 2017; Wilson et al., 2019). Second, the findings indicated 3DP models aided students in preparation for their *viva* assessment. Third, the results indicated that despite the condensed format of block model teaching, students report the use of the 3DP models as a positive learning tool that aid in knowledge acquisition and retention. These findings mirror those that have evaluated the use of models in the traditional semester model. Finally, the study offers a culturally sensitive and more economical alternative to anatomy education than the use of cadaver models.

## NOTES ON CONTRIBUTORS

NICHOLAS TRIPODI, B.Sc., M.Hsc., is a teaching focused academic at Victoria University in Melbourne, Australia. He teaches first‐year osteopathy students in clinical skills and anatomy. He is also a practicing osteopath, with a strong interest in running‐related injuries.

KATE KELLY, B.Psych. (Hons.), Ph.D., is a teaching focused academic at Victoria University in Melbourne Australia. She teaches first‐year psychology and communication units to psychology, social work, nutrition, and outdoor education students. She has a strong interest in cognitive neuropsychology in particular visual perception and memory.

MAJA HUSARIC, M.D., is an academic teaching researcher at Victoria University in Melbourne, Australia. She teaches first‐year bioscience to osteopathic, paramedic, and biomedical science students and has a special interest in cell biology and phototherapy.

REBECCA WOSPIL, B.Sc., M.Hsc., is a teaching focused academic at Victoria University in Melbourne, Australia. She teaches first‐year osteopathy students in clinical skills, anatomy, and research methods. She is also a practicing osteopath, with a keen interest in pain management.

MICHAEL FLEISCHMANN, B.Sc., P.G.Dip.Ex.Sci., G.C.T.E., M.Ost., is a teaching focused academic at Victoria University in Melbourne, Australia. He teaches research literacy skills and clinical skills to osteopathy students and is also a part time practicing osteopath.

SUSAN JOHNSTON B.Sc., M.Hsc., is a teaching focused academic at Victoria University in Melbourne, Australia. She specializes in teaching first‐year osteopathy students in clinical skills and anatomy.

KATHERINE HARKIN, B.Sc., M.Hsc., is a teaching focused academic at Victoria University in Melbourne, Australia. She teaches first‐year osteopathy students in clinical skills, anatomy, and communication methods and is also a practicing osteopath, with a keen interest in shoulder pain.

## Qualitative Survey and Focus Group Questions

Qualitative Survey Questions

- Did the three‐dimensional printed (3DP) models help/change your learning in this unit, inside and/or outside of class time?
- How did the 3DP models help you review material and prepare for the *viva* assessment?
- What effects do you think the 3DP models will have on your *viva* assessment performance and overall performance in the unit?
- Do you have any further comments/questions you would like to add regarding the 3DP models?

Focus Group Questions

- How did the 3DP models help me review material and prepare for the *viva* assessment?
- How did the 3DP models change your study habits both inside and outside of class?
- What effects did the 3DP models will have on your *viva* assessment performance and overall performance in the unit?
- Do you have any further comments you would like to add regarding the 3DP models?
